# Determinants of clinical outcome and revision surgery in spinal tumors: Analysis of 344 procedures

**DOI:** 10.1016/j.bas.2026.106089

**Published:** 2026-05-05

**Authors:** Fabian Winter, Astrid Dunst, Eric Freund, Magnus Kuess, Karl Roessler

**Affiliations:** Department of Neurosurgery, Medical University of Vienna, Vienna, Austria

**Keywords:** Spinal tumors, Surgical outcomes, Revision surgery, Perioperative complications, Intraoperative neuromonitoring

## Abstract

**Introduction:**

Spinal tumors present diagnostic and surgical challenges due to their anatomical and histological heterogeneity. Reliable determinants of postoperative outcome and revision surgery remain limited across mixed tumor cohorts.

**Research question:**

Which clinical, tumor-related and surgical factors predict postoperative neurological outcome and the need for revision surgery in patients undergoing spinal tumor resection?

**Material and methods:**

Single-center retrospective cohort of 344 resections in 326 patients during a 10-year period. Tumors were classified by anatomical compartment located intradural-extramedullary (45.3%), extradural (29.7%) or intramedullary (25.0%). Clinical outcome was defined as postoperative neurological outcome and revision surgery. Multivariate logistic regression was used to identify determinants of outcome.

**Results:**

Neurological improvement or stability was achieved in 84.6% of cases, with the best outcomes in intradural extramedullary tumors (90.3%). Revision surgery was required in 9.0% of cases. Gross total resection (GTR) was achieved in 71% and was the strongest positive determinator of favorable outcome (OR 1.9, p = 0.0266), while perioperative complications significantly reduced the likelihood of improvement (OR 0.6, p = 0.0230). Malignant histology and subtotal resection were associated with higher revision rates. Intraoperative neuromonitoring (IONM) showed a non-significant trend toward improved outcomes but was used more frequently in high-risk cases. Significant IONM deterioration occurred in 5.3% correlating with poorer long-term outcomes despite not determining immediate postoperative deterioration.

**Discussion and conclusion:**

Surgical outcome in spinal tumor patients is primarily determined by achieving safe GTR and minimizing perioperative complications. Tumor histology and anatomical location significantly influence prognosis. IONM remains valuable in complex cases.

## Key Points

• Tumor histology and anatomical location are strong predictors of surgical outcomes in spinal tumor cases.
• Gross total resection is significantly associated with better neurological improvement and lower revision rates.
• Perioperative complications are the most significant negative prognostic factor for clinical recovery.
• Revision surgeries were more frequent in cases of subtotal resection, metastases, and longer operative time.
• IONM signal change strongly predicts worse postoperative outcome, with a highly significant association (χ^2^ = 26.89, p = 6.2 × 10^−6^)

## Teaser

This retrospective study of 344 spinal tumor surgeries identifies histology, tumor location, resection extent, and perioperative complications as key predictors of clinical outcome and revision surgery. The findings support tailored surgical strategies and early risk identification in managing spinal neoplasms.

## 1 Introduction

Spinal tumors represent approximately 5–10% of all central nervous system neoplasms and pose significant diagnostic and therapeutic challenges due to their anatomical complexity and heterogeneous biological behavior. These lesions, which can be intramedullary, intradural extramedullary, or extradural, vary widely in prognosis and neurological impact depending on tumor type, location, and growth characteristics. Although surgical resection remains the mainstay of treatment for most primary and select metastatic spinal tumors, predicting clinical outcome and the likelihood of postoperative revision remains difficult.

Numerous factors have been proposed to influence surgical success in spinal tumor cases, including histological subtype, spinal compartment involvement, the extent of resection, and perioperative complications. However, previous studies often focus on specific tumor types or locations, limiting the generalizability of their findings. Additionally, large-scale cohort analyses that include a broad spectrum of spinal tumor entities and operative techniques remain sparse, particularly those that stratify by long-term clinical and revision outcomes. Analyzing a mixed-compartment cohort is of value as it reflects the real-world clinical decision-making in spinal oncology, where surgical principles like gross total resection and complication avoidance must be balanced across diverse histological entities.

The objective of this retrospective study was to identify associations between clinical, tumor-related, and surgical factors, specifically anatomical compartment, histology, extent of resection, and the use of IONM, with postoperative clinical outcomes and the necessity for revision surgery. We hypothesized that the extent of resection and the occurrence of perioperative complications serve as the primary independent determinants of neurological recovery and surgical success. By analyzing a consecutive series of 344 procedures, we aimed to determine the independent prognostic weight of these variables to provide a data-driven basis for surgical risk stratification and perioperative management in spinal oncology.

## 2 Materials and methods

This retrospective cohort study was conducted at the Department of Neurosurgery, Medical University of Vienna. Ethical approval was obtained from the institutional review board (IRB number: EK Nr. 1570/2021), and all procedures were carried out in accordance with the Declaration of Helsinki. Due to the retrospective design of the study, the requirement for informed consent was waived.

We reviewed all patients who underwent surgical treatment for spinal tumors between January 2011 and December 2021. To ensure a consecutive patient series and minimize selection bias, cases were identified through a systematic search of the institutional neurosurgical database using standardized diagnostic and procedural codes, followed by a detailed manual verification of all surgical records. Inclusion criteria were a histologically confirmed spinal tumor and the availability of complete perioperative and follow-up documentation. Patients who received only biopsies or non-surgical interventions were excluded. A total of 344 tumor resections in 326 patients met the inclusion criteria and were analyzed.

Data were extracted from the institutional neurosurgical database and verified through detailed chart review. The information collected included patient demographics, tumor location and histology, spinal level, anatomical compartment, operative details such as surgical approach, use of intraoperative neuromonitoring (IONM), extent of resection, as well as perioperative complications. Postoperative clinical outcome was determined based on documented neurological improvement or stability, and revision surgery was identified as any subsequent operation at the same spinal level related to tumor recurrence, residual tumor, or procedure-related complications.

Tumors were categorized according to their anatomical location, spinal level, and histopathological diagnosis. Anatomically, lesions were classified as intradural intramedullary (IDIM) when located within the spinal cord, intradural extramedullary (IDEM) when within the dura but outside the cord, and extradural (ED) when located outside the dura, including epidural or vertebral body involvement. Tumors were further grouped by spinal level into cervical, thoracic, lumbar, sacral, or multifocal when affecting non-contiguous regions. Benign tumors included meningiomas, schwannomas, ependymomas, and hemangioblastomas. Malignant primary tumors included gliomas and sarcomas. Metastatic tumors were categorized according to their primary origin (e.g., lung, breast, prostate, or renal cell carcinoma). Ambiguous or mixed histological subtypes were reviewed individually by board-certified neuropathologists.

All surgeries were performed under general anesthesia. The patient was positioned in the prone or lateral decubitus position depending on the location of the tumor and the surgical approach required. The posterior midline approach was the most employed technique, especially for IDEM and IDIM tumors. Hemilaminectomy or laminectomy was selected based on tumor extent, while laminoplasty was occasionally performed in younger patients or when multi-level access was required. Anterior or anterolateral approaches were reserved for certain cervical or thoracic ED tumors with vertebral body involvement.

Microsurgical resection under the operating microscope was standard in IDIM and most IDEM cases, often utilizing an ultrasonic aspirator (CUSA). The choice of dissection technique was guided by the tumor's consistency, vascularity, and proximity to neural structures. Gross total resection (GTR) was defined as complete removal of the contrast-enhancing tumor confirmed by early postoperative MRI (within 72 h). Subtotal resection (STR) was defined as resection of 80% or more of the tumor, leaving residual tissue where critical structures were at risk. Biopsy or partial debulking, defined as less than 80% removal, was documented in cases where total excision was not feasible.

IONM was utilized in 131 of the 344 procedures (38,08%) to improve surgical safety, especially in tumors involving or adjacent to the spinal cord. IONM was primarily indicated in cases with intramedullary tumors, tumors located in the high cervical region, lesions tightly adherent to neural tissue, or reoperations in which scar tissue complicated dissection. While these indications followed institutional recommendations for high-risk procedures, the final decision to utilize IONM was made by the lead surgeon, reflecting clinical judgment in individual cases. Modalities included somatosensory-evoked potentials (SSEPs) to evaluate dorsal column integrity, motor-evoked potentials (MEPs) to monitor corticospinal tracts, and electromyography (EMG), both spontaneous and triggered, to assess motor root function. D-wave monitoring was available for select high-risk IDIM tumors but was not used routinely. As is standard at our institution, a licensed and trained neurophysiologist was present in the operating room to closely monitor intraoperative neurophysiological data. Throughout the 10-year study period, alert criteria (50% amplitude drop) and the core use of SSEPs and MEPs remained standardized to ensure longitudinal consistency. A decrease of more than 50% in SSEP or MEP amplitude prompted immediate reassessment of surgical strategy, including cessation of dissection, irrigation, and optimization of hemodynamic parameters. Although the use of IONM did not show statistically significant impact in multivariate outcome analysis, a trend toward better postoperative results was noted.

Clinical outcome was defined as postoperative neurological improvement or stability when compared to preoperative status, as documented in follow-up assessments. To this end, all documentation regarding the patients’ general condition and clinical neurological status, as recorded by the treating physicians in discharge summaries or during outpatient visits, was reviewed. Revision surgery was defined as any reoperation at the same level for reasons including tumor recurrence, residual tumor burden, or postoperative complications.

All statistical analyses were performed using IBM SPSS Statistics, Version 27 (IBM Corp., Armonk, NY, USA). Continuous variables were expressed as mean ± standard deviation or median with interquartile range, depending on distribution. Categorical variables were reported as frequencies and percentages. Between-group comparisons were conducted using Chi-square or Fisher's exact tests for categorical variables, and Student's t-test or Mann-Whitney *U* test for continuous variables. Missing data were handled by case-wise exclusion from the respective statistical analyses.

To identify independent predictors of clinical outcome and revision surgery, multivariate logistic regression models were constructed. Variables were selected for inclusion based on clinical relevance and a significance level of p < 0.1 in univariate analysis. To ensure model stability and avoid overfitting, particularly regarding lower-frequency events such as revision surgeries, the number of predictors was kept parsimonious. Potential multicollinearity between categorical variables, such as tumor histology and anatomical compartment, was addressed by ensuring distinct clinical classification for each case to maintain the independence of predictors. Multicollinearity was formally assessed using Variance Inflation Factors (VIF), with values < 2.5 considered acceptable to maintain the independence of predictors. A p-value of less than 0.05 was considered statistically significant.

## 3 Results

**Fig. 1:**
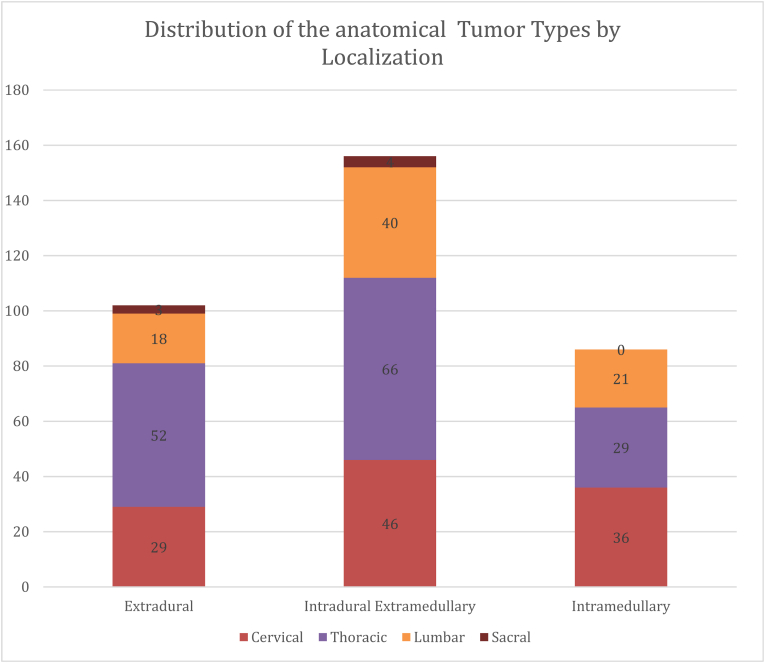
Distribution of spinal tumors by anatomical compartment.

**Fig. 2:**
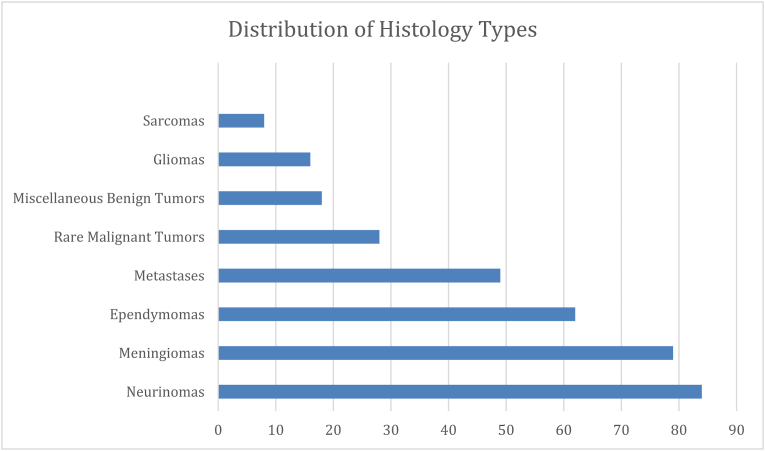
Distribution of histology types.

A total of 344 spinal tumor resections were performed in 326 patients. Most tumors were intradural extramedullary (45.3%), followed by extradural tumors (29.7%) and intramedullary tumors (25.0%) (Fig. 1). Meningiomas and schwannomas represented the largest histological subgroups (Fig. 2).

**Fig. 3:**
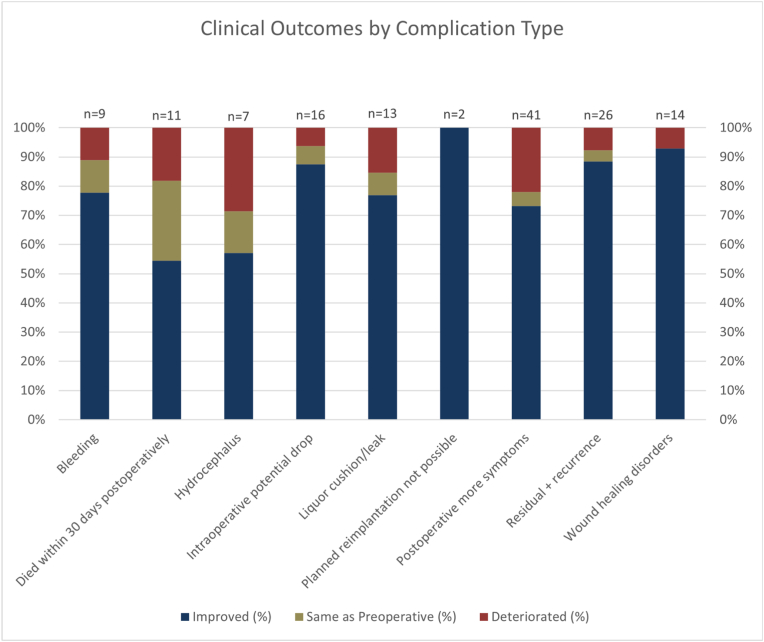
Clinical outcomes by complication type.

Clinical outcomes were evaluable in 333 of 344 surgeries. Improvement in neurological symptoms was observed in 291 cases, corresponding to an overall improvement rate of 84.6%. A small proportion of patients experienced deterioration (4.9%) or no change (7.3%) in their preoperative condition, while in 3.2% of cases, retrospective assessment of outcome was not possible (Fig. 3). The highest improvement rate was observed in patients with intradural extramedullary tumors (90.3%), followed by those with intramedullary tumors (81.6%) and extradural tumors (78.4%).

Surgical technique had a measurable influence on postoperative outcome. Hemilaminectomy yielded the highest improvement rate at 91.2%, followed closely by laminoplasty (86.4%). Laminectomy was associated with a lower improvement rate of 82.6%. The full breakdown by approach and monitoring is detailed in Table 1.

**Table 1:** Surgical outcomes by approach and monitoring.

Surgical Approach/Monitoring	Improved	Worsened	Same	Not Assessable (% of entire cohort)
Laminotomy	43 (81.1%)	3 (5.66%)	5 (9.43%)	2 (3.77%)
Laminectomy	128 (82.6%)	6 (3.87%)	12 (7.74%)	7 (4.52%)
Hemilaminectomy	52 (91.2%)	2 (3.5%)	1 (1.8%)	2 (3.5%)
Laminoplasty	19 (86.4%)	2 (9.09%)	0 (0%)	0 (0%)
Interarcuate Access	16 (80%)	1 (5%)	3 (15%)	0 (0%)
Far-lateral + Hemilaminectomy	8 (80%)	2 (20%)	0 (0%)	0 (0%)
Resection	18 (81.8%)	0 (0%)	4 (18.2%)	0 (0%)
Other	7 (87.5%)	1 (12.5%)	0 (0%)	0 (0%)
Without Monitoring	177 (83.1%)	8 (3.8%)	19 (8.9%)	9 (4.2%)
With Monitoring	114 (87.0%)	8 (6.9%)	6 (4.6%)	2 (1.5%)

**Table 2:** Multivariate analysis of outcome predictors.

Variable	Estimate	p-value	Significance
Successful Resection	+1.182	0.0266	Significant
Complications	−0.811	0.0230	Significant
Hemilaminectomy	+1.637	0.1072	Not significant
Intraoperative Monitoring	+0.423	0.3328	Not significant
Preoperative Radiation	−0.093	0.8376	Not significant

Patients without complications demonstrated an improvement rate of 88.6%, whereas those who experienced complications improved in only 75.4% of cases (see Table 2). Similarly, planned and successfully completed resections were associated with an improvement rate of 91.3%, while unsuccessful planned resections showed a substantially lower rate of 76.9% (Fig. 3).

Revision surgery was required in 9.01% of cases (n = 31). Consistent with our univariate findings, subtotal resection, malignant histology (e.g., metastases), and longer operative times were the primary factors associated with reoperation. The timing of these revisions was heavily skewed: while the mean interval to revision was 204 days, the median was significantly lower at 14 to 40.5 days for most indications, except for tumor recurrence, which showed a median of 40.5 days compared to a mean of 578 days. Surgeries requiring revision had a shorter mean operative time (194 min) compared to those that did not require revision (248 min).

The most frequent complications included cerebrospinal fluid leaks (3.8%) and wound healing disorders (4.1%). Postoperative neurological deterioration was reported in 12.3% of cases, while intraoperative potential changes occurred in 5.3%. A total of 12 patients (3.5%) died within 30 days postoperatively.

Statistical analysis showed that the frequency and type of complications varied significantly by histological tumor type. Gliomas and metastatic tumors had the highest complication rates, while meningiomas and schwannomas were associated with significantly lower rates.

Intraoperative neuromonitoring was employed to mitigate surgical risks. However, cases involving IONM showed a higher overall complication rate of 42.0%, compared to 29.1% in unmonitored procedures. This finding likely reflects selection bias. IONM was more frequently utilized in technically complex or high-risk surgeries where the baseline risk of complications is inherently higher. Ultimately, despite this perceived discrepancy, the data showed that the use of IONM was associated with a slightly higher rate of clinical improvement (87.0%) than procedures without IONM (83.1%), although this difference did not reach statistical significance in the multivariate analysis.

Multivariate logistic regression was used to identify independent predictors of clinical improvement, incorporating tumor compartment, histology, resection extent, IONM use, and perioperative complications into the model. Successful planned resection (GTR) was the strongest positive predictor with an odds ratio (OR) of 1.9 (95% CI: 1.1-3.4; p = 0.0266). Conversely, the occurrence of perioperative complications significantly reduced the likelihood of improvement (OR 0.6, 95% CI: 0.4-0.9, p = 0.0230). Preoperative irradiation also showed a negative trend (OR 0.63, 95% CI: 0.3-1.2), though it was not a significant independent factor in this cohort (p = 0.8376). While IONM use demonstrated a modest positive trend (OR 1.22, 95% CI: 0.7-2.1), it did not reach statistical significance (p = 0.3328).

**Fig. 4:**
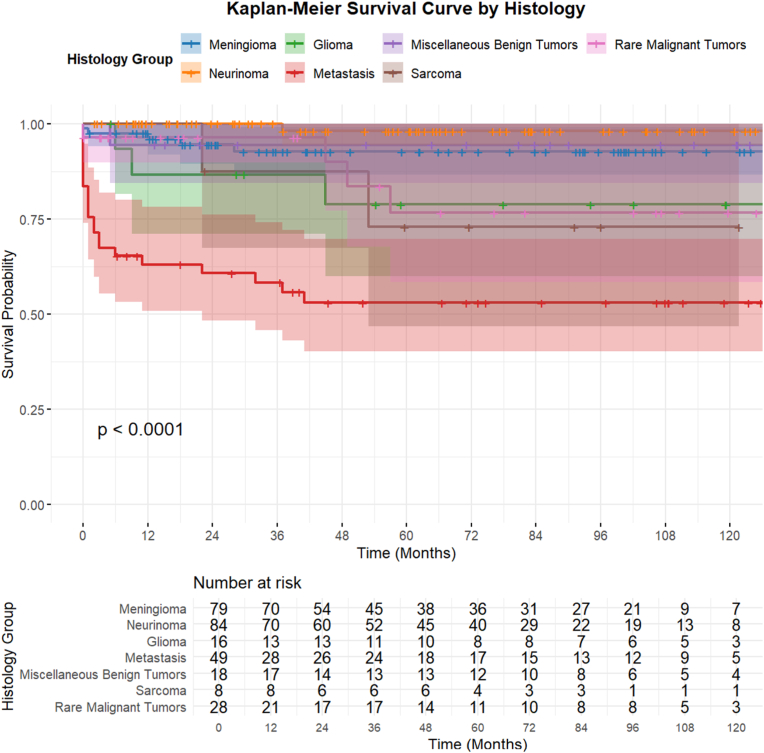
Kaplan-meier overall survival (OS) curve stratified by histology categories.

Meningiomas and schwannomas were associated with favorable postoperative recovery, with improvement rates exceeding 85% and minimal deterioration. Conversely, gliomas, metastases, and sarcomas demonstrated lower rates of clinical improvement and a higher likelihood of postoperative symptom worsening. Kaplan–Meier analysis stratified by histological tumor type demonstrated significantly different survival trajectories, with metastatic lesions showing the poorest survival and benign tumors such as meningiomas and schwannomas exhibiting favorable long-term survival (Fig. 4).

Intraoperative drop of IONM potentials was documented in 18 of 341 operations (5.3%). These events were significantly associated with prolonged surgical durations, with a mean operative time of 260 min, exceeding the average for other intraoperative complications. These losses or reductions in the signals derived during IONM will henceforth be reported in the manuscript as significant IONM signal changes (Table 3)

**Table 3:** Indications and timing for revision surgery.

Reason	Mean Days	Median Days	Min Days	Max Days	Total Cases	Percentage of Revisions (%)	Total Percentage of entire cohort (%)
Other (combined reasons)	34.6	14	0	195	9	25.00	2.62
Bleeding	41.9	9	0	195	8	22.22	2.33
Liquor fistula	2.25	2.5	0	4	8	22.22	2.33
Tumor residue or recurrence	578	40.5	0	3154	5	13.89	1.45
Progression of symptoms	17.8	13	9	28	4	11.11	1.16
Wound healing disorder	598	598	0	1196	2	5.56	0.58

No significant association was found between IONM signal change and postoperative symptom exacerbation at discharge (χ^2^ = 2.6845, p = 0.1013). Importantly, 87.5% of patients who experienced a signal drop showed clinical improvement at long-term follow-up. This suggests that while signal changes identify high-risk surgical phases, immediate intraoperative adjustments successfully mitigated the risk of permanent injury in most cases. In patients without IONM signal changes (n = 97), 40.2% exhibited increased postoperative symptoms. In contrast, among those with signal changes (n = 18), the rate was lower at 16.7%.

While these signal changes occurred in technically demanding cases, they did not result in permanent deficits in the majority of patients. At follow-up, a highly significant association was identified between IONM signal change and poor clinical outcome (χ^2^ = 26.89, df = 3, p = 6.2 × 10^−6^). Among the affected patients, 87.5% showed postoperative clinical improvement, while 6.25% showed no improvement and 6.25% remained clinically unchanged.

In logistic regression analysis, IONM signal change was associated with increased odds of prolonged hospitalization exceeding 14 days (odds ratio = 2.329, 95% confidence interval: 0.865–6.085; p = 0.084), although this result did not reach statistical significance. The association with admission to the intensive care unit was similarly not significant (odds ratio = 1.433, 95% confidence interval: 0.546–3.757; p = 0.458).

## 4 Discussion

This retrospective cohort study performed over a ten-year period and identified several key factors associated with clinical outcomes and the need for revision surgery in spinal tumors. Our findings underscore the critical influence of tumor histology, anatomical location, extent of resection, and perioperative complications on postoperative recovery. While prior studies have examined specific tumor subtypes or surgical techniques, this study offers one of the more comprehensive analyses to date across a broad histological and anatomical spectrum, including both primary and metastatic spinal neoplasms.

Clinical outcomes were evaluable in 333 of 344 surgeries. Among the assessable cases, neurological status improved in 291 procedures (87.4%), remained unchanged in 25 (7.5%), and worsened in 17 (5.1%). In 11 procedures (3.2%), retrospective assessment of outcome was not possible (Fig. 3). The highest improvement rate was observed in patients with intradural extramedullary tumors (90.3%), followed by those with intramedullary tumors (81.6%) and extradural tumors (78.4%). Surgical technique had a measurable influence on postoperative outcome. Among assessable cases, hemilaminectomy yielded the highest improvement rate (94.5%), followed by laminoplasty (90.5%).

The role of intraoperative neuromonitoring (IONM) in reducing complications remains a topic of ongoing debate. In our study, IONM did not significantly impact revision rates and was paradoxically associated with a higher overall complication rate. However, this likely reflects selection bias, as IONM was more frequently employed in technically complex or high-risk surgeries, in which complications would likely have occurred regardless of whether monitoring was used. This nuance is supported by Ibrahim et al. (2017) (Ibrahim et al., 2017), who noted that the benefits of IONM are most evident in challenging surgical cases and may not show uniform efficacy across all tumor types (Ibrahim et al., 2017). IONM signal change was not significantly associated with immediate postoperative symptom worsening, as patients with signal loss showed lower rates of acute deterioration compared to those without. However, a statistically significant correlation was observed between IONM signal change and poor long-term clinical outcomes, including a higher rate of unchanged or worsened neurological status. Summarized, Furthermore, a significant signal change can be viewed as a proxy for overall surgical trauma in high-risk cases where the cumulative effects of mechanical manipulation and microvascular compromise, though not resulting in immediate plegia, lead to a failure of long-term improvement compared to patients with stable intraoperative signals (Frisk et al., 2025). These events occurred predominantly in complex procedures such as laminectomy and were associated with longer operative times, highlighting their potential as prognostic markers for adverse surgical outcomes. The observed association between the use of intraoperative neuromonitoring (IONM) and a higher overall complication rate should be interpreted as a reflection of selection bias rather than a failure of the method itself. IONM was preferentially utilized in cases with high surgical complexity, such as intramedullary lesions or tumors with significant cord compression, where the baseline risk for morbidity is inherently elevated. Recent studies support this finding, suggesting that IONM serves as a surrogate marker for surgical difficulty in high-volume centers even in other pathologies (Hamilton et al., 2011). Furthermore, the fact that 87.5% of patients with an intraoperative signal drop still experienced long-term clinical improvement underscores the efficacy of immediate intraoperative adjustments, such as stopping dissection or optimizing blood pressure. This 'rescue effect' after neurophysiological alerts has been documented as a key factor in preventing permanent deficits in complex spinal procedures (Sutter et al., 2007). Consequently, our data reinforce the role of IONM not merely as a safety tool, but as a critical diagnostic adjunct that enables safer resection in high-risk scenarios.

Most patients in our cohort experienced postoperative clinical improvement (84.6%), a rate consistent with previous reports ranging from 70 to 90% in high-volume centers (Ng et al., 2018; Alvarez-Crespo et al., 2024). Outcomes varied significantly by tumor compartment. Intradural extramedullary tumors, including schwannomas and meningiomas, were associated with the most favorable prognosis, reflecting their typically benign behavior, well-defined anatomical boundaries, and accessibility to complete surgical resection. In contrast, intramedullary and extradural tumors presented greater surgical challenges and correspondingly lower improvement rates. These findings align with the observations by Ottenhausen et al. (2019) (Ottenhausen et al., 2019) and Arnautovic and Arnautovic (2009) (Arnautovic et al., 2009), who emphasized the impact of anatomical location on neurological outcomes and surgical complexity (Ottenhausen et al., 2019; Arnautovic et al., 2009). However, the pursuit of GTR must be carefully balanced against the risk of permanent postoperative morbidity, particularly when tumors involve eloquent neural structures. In our cohort, subtotal resection (STR) was often a deliberate choice aimed at preserving neurological function in cases where safe planes of dissection could not be clearly established. This strategy aligns with the contemporary surgical philosophy that maximal safe resection should be prioritized over aggressive radicality to maintain the patient's long-term quality of life (Moreau et al., 2020; Panigrahi et al., 2024). Such a nuanced approach remains especially critical in intramedullary lesions, where the margin between oncological control and irreversible neurological injury is exceptionally narrow (Panigrahi et al., 2024). Consistent with our findings, prioritizing functional integrity over total removal in high-risk scenarios ensures that surgical intervention does not outweigh the natural history of the disease.

Perioperative complications emerged as the most robust negative predictor of postoperative recovery. Patients experiencing complications demonstrated a significantly reduced improvement rate and were more likely to require extended hospitalization and ICU care. The most common complications included neurological deterioration, CSF fistula, wound healing disorders, and bleeding. These findings reinforce those of Lange et al. (2022) (Lange et al., 2022), who identified perioperative complications and prolonged ICU stays as key contributors to poor outcomes in spinal surgery (Lange et al., 2022).

Our study also highlights the detrimental effect of preoperative irradiation, which was associated with both a reduced likelihood of clinical improvement and an increased rate of 30-day postoperative mortality. This observation aligns with the meta-analysis by Luksanapruksa et al. (2017) (Luksanapruksa et al., 2017), which demonstrated that radiation-induced tissue changes compromise wound healing and elevate complication rates. Feler et al. (2022) (Feler et al., 2022) further emphasized that irradiated tissues increase surgical complexity and the risk of delayed recovery (Luksanapruksa et al., 2017; Feler et al., 2022).

This study has several limitations. The principal limitation of this study is the reliance on documented physician assessments rather than standardized functional scales (e.g., McCormick or Modified Japanese Orthopaedic Association scores), which introduces potential ascertainment bias. Its retrospective design introduces inherent bias, including selection and documentation variability. Although our sample size is large compared to similar studies, subgroup analysis was limited for rare tumor types. Functional outcomes were primarily assessed through chart review rather than standardized scoring systems, data regarding other radiation oncology treatments were not available. Moreover, while multivariate analysis controlled for several key variables, residual confounding cannot be excluded. The generalizability of the results is also limited by the single-center nature of the study.

Despite these limitations, the study offers valuable insights into the prognostic factors influencing outcomes in spinal tumor surgery. It affirms the importance of achieving gross total resection whenever safely feasible, minimizing perioperative complications, and considering histological subtype in both risk stratification and surgical planning. Future prospective studies with standardized functional assessments, standardized scales and long-term follow-up are warranted to refine prognostic models and enhance outcome prediction.

Additionally, the findings underline the prognostic relevance of intraoperative neuromonitoring signal loss, particularly drops in signal potential, which were strongly associated with poor long-term neurological outcomes. While some studies question the overall predictive value and cost-effectiveness of neuromonitoring in reducing complications and revisions (Ibrahim et al., 2017; Ament et al., 2023), others emphasize its role in enabling timely intraoperative adjustments and improving outcomes when applied with appropriate expertise (Kothbauer, 2007; Nuwer et al., 1995; Abdelmalek et al., 2024).

## 5 Conclusion

In conclusion, surgical success is primarily driven by meticulous complication avoidance and the achievement of safe gross total resection. While IONM signal changes are not direct predictors of permanent deficits, they serve as critical markers of high surgical complexity, necessitating immediate intraoperative adjustment to optimize patient outcomes.

## Statement on AI use

ChatGPT (OpenAI, San Francisco, USA) was used for language optimization throughout the manuscript.

## Conflicts of interest

None declared.
